# Nanotheranostics With the Combination of Improved Targeting, Therapeutic Effects, and Molecular Imaging

**DOI:** 10.3389/fbioe.2020.570490

**Published:** 2020-09-15

**Authors:** Shin-Lei Peng, Chih-Ho Lai, Pei-Yi Chu, Jer-Tsong Hsieh, Yen-Chun Tseng, Shao-Chieh Chiu, Yu-Hsin Lin

**Affiliations:** ^1^Department of Biomedical Imaging and Radiological Science, China Medical University, Taichung, Taiwan; ^2^Department of Microbiology and Immunology, Molecular Infectious Disease Research Center, Chang Gung University, Chang Gung Memorial Hospital, Taoyuan, Taiwan; ^3^Faculty of Pharmacy, National Yang-Ming University, Taipei, Taiwan; ^4^Department of Urology, University of Texas Southwestern Medical Center, Dallas, TX, United States; ^5^Department of Biological Science and Technology, China Medical University, Taichung, Taiwan; ^6^Center for Advanced Molecular Imaging and Translation, Chang Gung Memorial Hospital, Taoyuan, Taiwan; ^7^Department of Medical Research, China Medical University Hospital, China Medical University, Taichung, Taiwan; ^8^Institute of Biopharmaceutical Science, Department and Institute of Pharmacology, Center for Advanced Pharmaceutics and Drug Delivery Research, National Yang-Ming University, Taipei, Taiwan

**Keywords:** targeting tumors, fucoidan, hyaluronic acid, molecular images, nanotheranostics

## Abstract

There is an increasing interest in the design of targeted carrier systems with combined therapeutic and diagnostic modalities. Therapeutic modalities targeting tumors with single ligand-based targeting nanocarriers are insufficient for proficient delivery and for targeting two different surface receptors that are overexpressed in cancer cells. Here, we evaluated an activated nanoparticle delivery system comprising fucoidan/hyaluronic acid to improve therapeutic efficacy. The system comprised polyethylene glycol-gelatin-encapsulated epigallocatechin gallate (EGCG), poly (D,L-lactide-co-glycolide; PLGA), and stable iron oxide nanoparticles (IOs). The latter enables targeting of prostate cancers in their molecular images. We demonstrate the transfer of nanoparticles and their entry into prostate cancer cells through ligand-specific recognition. This system may prove the benefits of drug delivery that enhances the inhibition of cell growth through apoptosis induction. Moreover, the improved targeting of nanotheranostics significantly suppressed orthotopic prostate tumor growth and more accurately targeted tumors compared with systemic combination therapy. In the presence of nanoparticles with iron oxides, the hypointensity of the prostate tumor was visualized on a T2-weignted magnetic resonance image. The diagnostic ability of this system was demonstrated by accumulating fluorescent nanoparticles in the prostate tumor from the *in vivo* imaging system, computed tomography. It is suggested that theranostic nanoparticles combined with a molecular imaging system can be a promising cancer therapy in the future.

## Introduction

Cancer is a heterogeneous group of diseases characterized by the generation of abnormal cells. Such cells proliferate uncontrollably, invading and destroying normal tissues (Salako et al., 2017; Gao et al., 2019; Roma-Rodrigues et al., 2019). Current cancer therapies may not achieve the optimum prognosis. Thus, effective and innovative targeted therapies are required (Leach et al., 2016; Yang et al., 2019). Moreover, single-target drug carriers may be unsatisfactory for selective and effective therapy. Therefore, carriers targeting different surface receptors overexpressed in tumor tissues have been studied (Kos et al., 2015; Gamper et al., 2019). Cancer cells usually present multiple surface antigens. For example, overexpression of fucosylated epitopes, such as type I and type II Lewis antigens, frequently occurs on the surface of cancer cells, and these molecules are highly expressed in association with high levels of Lewisy in localized and metastatic prostatic adenocarcinoma (Myers et al., 1995; Blanas et al., 2018). Fucosylation occurs when fucose residues are transferred to an oligosaccharide chain attached to a cell surface glycoprotein or glycolipid (Ma et al., 2006). Fucoidan (FU), a polysaccharide containing numerous L-fucose and sulfate ester residues, is a fucosylated agent binding material and has been considered as a binding agent of P-selectin (Preobrazhenskaya et al., 1997). The upregulation of P-selectin on tumor blood vessels has been demonstrated in a previous study (Preobrazhenskaya et al., 1997). Chung et al. (2020) used dendrimer-fucoidan polyionic nanocomplex specifically targets triple-negative breast cancer overexpressing P-selectin and tumor-related vasculature.

Prostate cancer cells can spread to other body parts, particularly the bone, where they adhere strongly to bone marrow endothelial cells that show high levels of membrane-associated CD44 (Draffin et al., 2004; van der Toom et al., 2016; Gao et al., 2018). Stem cell-like CD44^+^ prostate cancer cells invade *in vitro* and metastasize *in vivo* (Draffin et al., 2004; Liu et al., 1999). The CD44 molecules expressed by cancer cells interact with hyaluronan (HA)-rich microenvironments, thereby affecting signaling pathways that induce malignant cells to invade and migrate, initiating metastatic tumor cell inflammation (Park et al., 2008; Chen et al., 2017). HA is a linear glycosaminoglycan comprising alternating disaccharide units of N-acetyl-D-glucosamine and D-glucuronic acid with β(1 → 4) interglycosidic linkages (Mizrahy et al., 2011). In addition, HA can be used as a targeting molecule toward cancer cells in nanoparticle (NP) delivery systems and increase the cellular uptake of NPs (Mattheolabakis et al., 2015). The numerous strategies that specifically target cancer stem cells have met with limited success (Krishnamurthy et al., 2015). NPs specifically and effectively target such cells (Bae et al., 2018; Deng et al., 2020). Poly (D,L-lactide-co-glycolide; PLGA) is commonly used in NPs employed in biomedical applications because of its excellent biocompatibility and is approved by the United States Food and Drug Administration (Sah and Sah, 2015). However, intravenously administered PLGA NPs, like other conventional colloidal carriers, are rapidly removed from the circulation by macrophages (Avgoustakis, 2004). Polyethylene glycol (PEG), a water-soluble polymer, can modify gelatin [PEG-gelatin (PG)] to decrease the cytotoxicity of doxorubicin significantly and increase the stability of NPs in cancer treatment (Lee et al., 2006). For example, Cao et al. (2016) used PEGylated PLGA NPs to prolong circulation time by preventing opsonin binding, which reduces the rapid uptake of NPs by the reticuloendothelial system.

**FIGURE 1:**
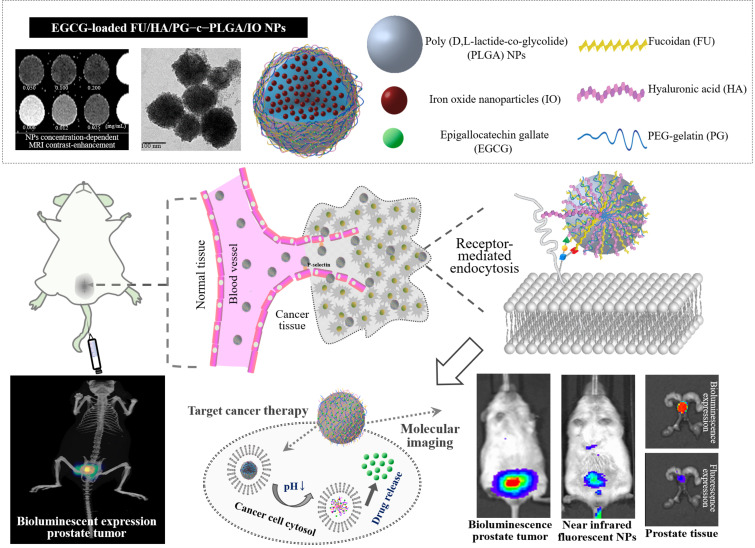
Schematic representation of using an effective epigallocatechin gallate (EGCG)-loaded FU/HA/PG-coated [Poly (D,L-lactide-co-glycolide); PLGA] nanoparticles (NPs) targeting strategy and observation of their putative effects on prostate carcinoma cells.

The use of combination therapies targeting the metabolic and physiological properties of cancer cells reduces drug resistance. However, differences in their pharmacokinetics, inconsistency of drug uptake by tumor cells, and suboptimal drug concentrations *in vivo* limit their efficacy in cancer therapy (Park et al., 2008; Frohlich, 2012). Epigallocatechin gallate (EGCG), a phytochemical extracted from green tea, binds with high affinity to laminin receptors, which are overexpressed in prostate cancer cells. Furthermore, EGCG inhibits matrix metalloproteinases, which are associated with tumor invasion and metastasis, inducing apoptosis in prostate cancer cells and prostate cancer stem cells (Shukla et al., 2012; Zhou et al., 2016). EGCG has prompted increased interest in the design of nanoagents with combined therapeutic and diagnostic properties (Chung et al., 2014; Cheng et al., 2016; Du et al., 2016). Moreover, the design of targeting carrier systems by combining therapeutic and diagnostic modalities is gaining increasing attention. Smart platforms of hybrid nanostructures to release chemotherapeutic molecules/drugs in response to an external stimulus to perform remotely controlled therapeutics, diagnostics, and therapy monitoring in a variety of solid tumors, including brain cancer, breast cancer, and osteosarcoma, have been reported (Li et al., 2019; Min et al., 2019; Su et al., 2020; Thorat and Bauer, 2020). To develop nanoagents with diagnostic properties, iron oxide nanoparticles (IOs) can be used as magnetic resonance imaging (MRI) contrast agents to produce high-resolution and high-contrast images of tissues (Weissleder et al., 1990; Weinstein et al., 2010; Luong et al., 2017; Xu et al., 2019). Here, we specifically developed NPs comprising PLGA-containing IOs for biological imaging, using FU/HA to achieve targeting activity and applying PG-carrying EGCG to eradicate prostate tumors (Figure 1). Its efficacies were examined in cancer cells and in an orthotopic tumor mouse model.

## Materials and Methods

### Materials

Hyaluronan and FU were purchased from Lifecore Biomedical, LLC (Chaska, MN, United States) and Chambio, Co., Ltd. (Taichung, Taiwan). Oleic-acid-coated IO nanoparticles (5 nm in diameter) dispersed in chloroform were obtained from AC Diagnostics, Inc. (Fayetteville, AR, United States). The N-hydroxylsuccinimide (NHS) functionalized methoxy polyethylene glycol (molecular weight 5,000 Da) was obtained from Nanocs, Inc. (New York, NY, United States). The VivoTag^®^-S 750 (VT750) is an amine reaction (NHS ester) near-infrared fluorochrome (excitation/emission at 750/775) purchased from PerkinElmer, Inc. (Waltham, MA, United States). Type A gelatin (molecular weight 25,000 Da), dimethyl sulfoxide, 3-(4,5-dimethylthiazol-2-yl)-2,5-diphenyltetrazolium bromide (MTT), rhodamine 6G (R6G), fluoresceinamine (FA), fluorescein isothiocyanate (FITC), 4′,6-diamidino-2-phenylindole (DAPI), phosphate-buffered saline (PBS), puromycin, EGCG, and Triton X-100 were purchased from Sigma-Aldrich (St Louis, MO, United States). Fetal bovine serum (FBS), Roswell Park Memorial Institute Medium, penicillin-streptomycin were from Gibco (Brooklyn, New York, United States). All other chemicals and reagents were of analytical grade.

### Preparation and Characterization of FHP-c-PLGA NPs

Poly (D,L-lactide-co-glycolide) nanoemulsion particles were produced using a water-in-oil emulsification method employing homogenization using a rotating blade homogenizer (IKA Labortechnik, Germany). A 2.0 mL volume of D-α-tocopheryl polyethylene glycol succinate (TPGS) surfactant solution (30.0 mg/mL) was poured slowly into ethyl acetate containing 2.0 mL of PLGA solution (4.0 mg/mL). The mixture was homogenized at 15,000 rpm for 5 min at 4°C. The prepared PLGA NPs were centrifuged, then the pellets were washed with distilled water to remove the organic solvent, and then the precipitate was collected and suspended in deionized water for used the rest of the FHP–c–PLGA NPs study. The solution (1.0 mL) of polyvinyl alcohol surfactant (48.0 mg/mL) and different FU:HA:PG compositions (0.0:0.0:0.0, 1.2:1.2:1.2, 2.4:2.4:2.4, and 4.8:4.8:4.8 mg/mL) were mixed into the aqueous PLGA NPs solution (2.0 mg/mL, 1.0 mL) through a pipette tip with gentle stirring and then allowed to react for 2 h to form FU/HA/PG–coated PLGA NPs (FHP–c–PLGA NPs). The NPs were centrifuged and suspended in deionized water for Fourier-transform infrared spectroscopy (FTIR) analysis or placed onto a 400 mesh copper grid for scanning electron microscopy.

### Characterization and Drug Release Profiles of EGCG-Loaded FHP-c-PLGA NPs

To study the release of EGCG from test NP samples, we prepared EGCG-loaded FHP–c–PLGA NPs. The EGCG solutions (0.0, 1.0, 2.0, 3.0 mg/mL; 1.0 mL) were each mixed with 1.0 mL of aqueous 2.0 mg/mL NPs and then stirred at room temperature. The NPs comprising PEG-gelatin were homogenized to form encapsulated EGCG. Gelatin is a water-soluble protein mixture derived from the natural polymer collagen (Boni et al., 2018; Chivere et al., 2020). Previous studies have shown that it can bind polyphenols via hydrogen-bonding interactions among amino acids such as proline and the phenol ring of polyphenols (Shutava et al., 2009; Mi et al., 2018). The EGCG loaded NPs were collected by centrifugation, and the concentration of free EGCG in the supernatant was determined using high-performance liquid chromatography (HPLC). The drug loading efficiency and loading content of NPs were described in the literature and calculated according to the following equation:

(1) $$\text{loading}\text{efficiency}=\frac{\text{total}\text{amount}\text{of}\text{EGCG}-\text{free}\text{EGCG}\text{in}\text{supernatant}}{\text{total}\text{amount}\text{of}\text{EGCG}}\times 100\%$$

(2) $$\text{loading}\text{content}=\frac{\text{total}\text{amount}\text{of}\text{EGCG}-\text{free}\text{EGCG}\text{in}\text{supernatant}}{\text{weight}\text{of}\text{NPs}}\times 100\%$$

To investigate the stability of EGCG-loaded NPs, NPs were incubated at pH 7.4, 6.5 (10 mM PBS) and then at pH 5.0 (10 mM acetic acid/sodium acetate), simulating the pH values of physiological fluids, tumor tissues, and endosomal compartments (Qiu et al., 2008; Tian et al., 2013; Nehate et al., 2017). The EGCG-loaded NPs (2.0 mg/mL, 0.2 mL) were added to dialysis bags and dialyzed against 2.0 mL of these solutions. The dialysates (0.1 mL) were sampled and replenished with the same freshly prepared buffers to prevent drug saturation. The drugs released into the dialysate were detected using an HPLC system to determine the percentage of cumulative drug release. NP morphology was examined using transmission electron microscopy (TEM).

### Cell Viability After Treatment With Only NPs and EGCG Solution or EGCG-Loaded NPs

For cytocompatibility studies, normal prostate epithelial cells (PZ-HPV7; ATCC^®^ CRL-2221^TM^) and human prostate cancer cells (PC3 cells; ATCC^®^ CRL1435) were seeded in a 96-well plate at a density of 1.0 × 10^4^ cells/well and maintained at 37°C and 5% CO_2_ for overnight. The media containing different concentrations of only FHP-c-PLGA NPs and the cells were incubated for 2 h, and cells were washed twice with PBS and cultured in fresh growth medium for 22 h. Cells exposed to media only served as positive controls. The cell viability was determined at each time point using cell viability MTT assays. Moreover, the prostate cancer cells were allowed to adhere overnight and then treated with the EGCG solution or EGCG-loaded NPs containing different concentrations of EGCG for 2 h. Cell culture supernatants were gently removed, and cultured in fresh growth medium for 22 h prior to evaluation of cellular toxicity with MTT assays. Changes in cell morphology induced by EGCG-loaded FHP–c–PLGA NPs or only FHP–c–PLGA NPs and control samples were analyzed using an inverted microscope under phase-contrast illumination (10 × objective; Olympus, Japan).

### Confocal Laser Scanning Microscopy Observation of Fluorescent-Labeled NP on Cancer Cells

To quantify the fluorescence signal of the NPs [R6G-FU/FA-HA/Cyanine 5 (Cy5)-PG–c–PLGA or Cy5-EGCG-loaded R6G-FU/FA-HA/PG–c–PLGA NPs] in cells, fluorescent FA-conjugated HA (FA-HA) was produced through reactions between FA (excitation/emission at 496/525) amine moieties and HA carboxylic acid groups based on earlier work carried out by Harada et al. (2006), with some modifications. R6G-FU was produced as R6G (excitation/emission at 515/555) in acetonitrile solution (1 mg/1 mL), added gently to FU in distilled water (100 mg/10 mL), and mixed with 1 mg of 1-ethyl-3-(3-dimethylaminopropyl) carbodiimide hydrochloride with continuous stirring for 12 h at 4°C. To eliminate unconjugated fluorescent R6G dye, R6G-FU was dialyzed against distilled water in the dark until no further fluorescence remained in the supernatant. The product was then lyophilized. Cy5-NHS or Cy5-hydrazide (excitation/emission at 646/662) was conjugated to PG or EGCG to prepare Cy5-PG or Cy5-EGCG, respectively. Cy5-NHS or Cy5-hydrazide in dimethyl sulfoxide (1 mg/mL) was prepared and added gradually to soluble PG or EGCG in distilled water (100 mg/10 mL) with continuous stirring for 12 h. To remove the unconjugated Cy5-NHS, Cy5-PG was dialyzed until fluorescence was undetectable in the supernatant. Cy5-EGCG was lyophilized using a freeze dryer, and lyophilized Cy5-EGCG was dissolved in distilled water. The precipitate was removed and subjected to repeated cycles of washing and centrifugation until the fluorescence of Cy5-hydrazide dye was undetectable in the precipitate. Cy5-PG and Cy5-EGCG were then lyophilized.

To detect the internalization of fluorescent NPs in PC3 cells, the cells were incubated on glass cover slip (3 × 10^5^ cells/cm^2^) for 1 day. Cells (2 × 10^5^) were incubated with a Global Eukaryotic Microcarrier^®^ (Global Cell Solutions, United States) in LeviTubes culture vessels at 37°C and 5% CO_2_ for 7 days to track the NPs internalization of three-dimensional cancer cells. Test samples (0.4 mg/mL) were added to cells, incubated for 2 h, aspirated and washed with PBS, and fixed in 3.7% paraformaldehyde. The cells were permeabilized with 2 mg/mL Triton X-100 and stained with DAPI and observed using a confocal laser scanning microscope (CLSM).

### Confocal Microscopy Observation of Apoptosis and Flow Cytometric Analysis of Cell Cycle

To analyze the EGCG-loaded NPs effects on apoptosis, FITC-Annexin V solution was used to detect apoptotic cells following the instructions provided by the manufacturer. The cells (3 × 10^5^ cells/cm^2^) were incubated on glass coverslips for 24 h. The test samples were put into cells for 2 h and were incubated in growth medium for further incubated 22 h. After washing with PBS and suspending in binding buffer, FITC-Annexin V was added to the cells and incubated for 15 min at 4°C, and stained cells were recorded by using confocal microscopy (excitation at 488 nm and emission at 525 nm; Gao et al., 2011). Subsequently, to examine the effects of EGCG-loaded NPs on cell cycle progression, PC3 cells were incubated with NP samples for 2 h, washed twice in PBS, and incubated in growth media for 22 h. Cells were washed with ice-cold PBS and then fixed in ice-cold 70% ethanol. After extensive washing, cells were suspended in hypotonic buffer and incubated at 37°C for 1 h. Propidium iodide (1.00 mg/mL, 0.01 mL) was added and then incubated for 0.5 h at 4°C in the dark. The propidium iodide and a BD FACSCanto System (BD Biosciences, United States) were used to analyze the cell cycle phases.

### Western Blotting Analysis of Apoptosis-Related Proteins

To evaluate the expression of apoptotic proteins, PC3 cells were subjected to western blotting analysis after treatment with EGCG-loaded NPs. For this purpose, cells were lysed using freeze/thaw cycles, and the cell lysates were centrifuged at 6,000 g at 4°C. Equal amounts of cell protein lysates were electrophoretically separated through sodium dodecyl sulfate (SDS) polyacrylamide gel, and the separated proteins were electrophoretically transferred onto polyvinylidene difluoride (PVDF) membranes, which were putted into PBS containing 5% (w/v) nonfat dry milk for 1 h and then probed with primary antibodies [i.e., rabbit poly-clonal anti-caspase-9 and anti-poly (ADP-ribose) polymerase (PARP)]; mouse monoclonal anti-caspase-8, anti-caspase-3 and anti-β-actin) overnight at 4°C. Membranes were incubated with horseradish peroxidase-conjugated secondary anti-bodies for 1 h, and immune complexes were detected enhanced chemiluminescence (Amersham Life Science, United States).

### Mouse Model of Orthotopic Prostate Tumor

Animal care and use is in accordance with the 1996 revision of the Guide for the Care and Use of Laboratory Animals issued by the National Research Council Laboratory Animal Resources Institute. Severe combined immunodeficiency (SCID) 6-week-old males were used for xenografting. The mice were housed in the animal facility at least 1 week before injection with luminescent PC3 cells (Luc-PC3 cells) from Professor Jer-Tsong Hsieh (UT Southwestern Medical Center, Dallas, TX). After the abdomens of SCID mice were sterilized with alcohol, a transverse incision was made into the lower abdomen and bladder, and the seminal vesicles and prostate were removed from the abdominal cavity to expose the anterior prostate. Luc-PC3 cells (3 × 10^6^/20 μL) were injected into the prostate gland. The bladder was replaced, and the muscle layer was closed using absorbable vicryl monofilament sutures. The skin layers were secured using sterile staples.

### Magnetic Resonance Imaging (MRI) and *in vivo* Imaging System-Computed Tomography (IVIS Spectrum CT) Detection of NPs in Tumors

To achieve MR-based molecular imaging of the prostate tumor, the FHP–c–PLGA-encapsulated IO (FHP-c-PLGA/IO NPs) were produced according to the procedure described in previous section. The mean particle sizes and zeta potential values of the FHP-c-PLGA/IO NPs were measured with a Zetasizer instrument. MR-T2 relaxation times of FHP-c-PLGA/IO NPs were determined using a 7T MRI system (Bruker ClinScan 70/30, Germany). Samples were diluted with distilled water before adding them to 96-well microplates. Relaxivity was calculated by acquiring a multiecho spin echo sequence as follows: repetition time (TR) = 2000 ms; echo time (TE) = 7.2–230.4 ms (7.2 ms intervals); slice thickness = 1 mm; matrix size = 256 × 256; field of view (FOV) = 50 × 50 mm. The images were analyzed using custom Matlab scripts (The MathWorks, United States). Spin–spin T2 relaxation was estimated as follows: *M* = M_0_e^–*TE*/*T*2^, where M_0_ is the magnetic moment at equilibrium. For *in vivo* MR imaging of FHP–c–PLGA/IO NPs, eight SCID mice were used. A prostate tumor model was established by injecting cancer cells into the prostates of recipients. Four other mice served as controls. All MRI experiments were conducted using a 7T animal MRI scanner (gradient strength = 630 mT/m). The volume coil was used for signal excitation and the surface coil for signal detection. Mice were anesthetized with 4% isoflurane, which as reduced to 2.5% isoflurane for maintenance. A 30-gage needle connected to a 0.8 m polyethylene-30 tube was inserted into the tail vein for the injection of FHP–c–PLGA/IO NPs. A T2-weighted (T2W) fast spin echo sequence was used before and after one hour sample injection as follows: TR = 1,780 ms; TE = 69 ms; slice thickness = 0.8 mm; matrix size = 208 × 256; FOV = 36 × 45 mm.

To further observe the distribution of NPs in Luc-PC3 cells in xenografts, near-infrared fluorescent VT750-labeled NPs were injected through the tail vein into Luc PC3 tumor-bearing mice. Briefly, 0.1 mL of 1.0 mg/0.1 mL VT750 dye solution in dimethyl sulfoxide was added gradually to 5.0 mL of 10.0 mg/mL soluble PG in distilled water with continuous stirring for 12 h at 4°C. The unreacted dye was removed via dialysis, and the VT750-conjugated PG (VT750-PG) was lyophilized. The near-infrared fluorescent FU/HA/VT750-PG–c–PLGA NPs was prepared using the same protocol as for the preparation of NPs. Fluorescence imaging was performed using an *in vivo* imaging system (IVIS; excitation 710–760 nm; emission 810–875 nm). CT images were acquired using an IVIS Spectrum CT scanner (PerkinElmer, Inc., United States) with the settings as follows: 50 kV at 1 mA, 140 s acquisition and reconstruction times, and aluminum filter. We acquired 720 projections spaced 0.5° apart, and the CT volume was reconstructed using Living Image software with a FOV = 12.0 cm × 12.0 cm × 13.0 cm at 0.15 mm isotropic resolution.

### Evaluations of Antitumor Activity, Bioluminescent Imaging, and Histological Analysis

To evaluate antitumor activity, the orthotopic luminescent prostate tumors were grown for 7 days. The SCID mice were then randomly divided into three groups of six mice each, which received different formulations of 0.1 mL of 15.0 mg EGCG/kg, EGCG-loaded FHP–c–PLGA NPs, and FHP–c–PLGA NPs (controls). Mice were injected in the tail vein every third day. Bioluminescent imaging *in vivo* was performed using an extremely sensitive charge-coupled device camera included with the IVIS Spectrum *in vivo* optical imaging system and viewed in real-time to measure total flux (pho-tons/s/cm^2^/steradian). The condition and body weight of each mouse was determined. One day after the last bioluminescent observation, the mice were sacrificed and their tissues were removed for histological examination. Tissue biopsies were performed with the aid of a visible-light microscope, and biopsies were stained with hematoxylin-eosin or subjected to immunohistochemistry using rabbit monoclonal antibodies against Ki-67 (Thermo Fisher Scientific, United States) and cleaved PARP (Cell Signaling Technology Inc., United States). Tissue inflammation and the microdistribution of stained tissue sections were detected at different magnifications using a visible light microscope.

### Statistical Analysis

Statistical analysis to assess differences between groups was performed using one-way analysis of variance. The Statistical Analysis System was used to calculate the Confidence intervals. Data are presented as the mean ± standard deviation. The *p* values with an asterisk indicating *p* < 0.05 and two asterisks indicating *p* < 0.01 are considered statistically significant.

## Results

### Preparation and Characterization of FHP-c-PLGA NPs

Poly (D,L-lactide-co-glycolide) nanoemulsion particles were prepared using a water-in-oil emulsification method with TPGS nonionic surfactant [hydrophile lipophilic balance (HLB) = 13.2]. Different concentrations of the FU/HA/PG mixture coated onto the PLGA NP solution were examined to find the optimal formulation for FU/HA/PG-coated PLGA NPs (FHP–c–PLGA NPs) preparation. As shown in Table 1, the mean particle size and polydispersity index of NPs were ranging from 158.64 ± 9.87 to 252.47 ± 19.76 nm and from 0.13 ± 0.04 to 0.43 ± 0.16, respectively. At a FU:HA:PG ratio of 1.2:1.2:1.2 mg/mL, NPs produced a particle size of 198.89 ± 12.56 nm with a negative zeta potential value of -33.75 ± 1.24 mV and had a significantly narrower distribution (0.22 ± 0.09). However, when the FU:HA:PG concentration was elevated to 2.4:2.4:2.4 mg/mL, the NP polydispersity index was increased to 0.4, indicating high heterogeneity. The polydispersity index is a parameter used to define particle size distribution and is a dimensionless number extrapolated from the autocorrelation function that ranges from 0 to 1. Values close to 0 indicate a homogeneous dispersion, while those greater than 0.3 indicate high heterogeneity. Therefore, the optimal FU:HA:PG concentrations were 1.2:1.2:1.2 mg/mL which therefore be used in subsequent experiments.

**TABLE 1:** Effect of different FU/HA/PG concentrations on particle sizes, polydispersity indices, and zeta potential values of the FHP-c-PLGA NPs (*n* = 5).

FU:HA:PG (mg/mL)	Mean Particle Size (nm)	Polydispersity Indices	Zeta Potential Value (mV)
			
0.0:0.0:0.0	158.64 ± 9.87	0.13 ± 0.04	−28.76 ± 2.47
0.6:0.6:0.6	177.57 ± 7.98	0.17 ± 0.06	−30.87 ± 1.78
1.2:1.2:1.2	198.89 ± 12.56	0.22 ± 0.09	−33.75 ± 1.24
2.4:2.4:2.4	252.47 ± 19.76	0.43 ± 0.16	−36.37 ± 3.87

*PLGA, Poly (D,L-lactide-co-glycolide); NPs, nanoparticles.*

**FIGURE 2:**
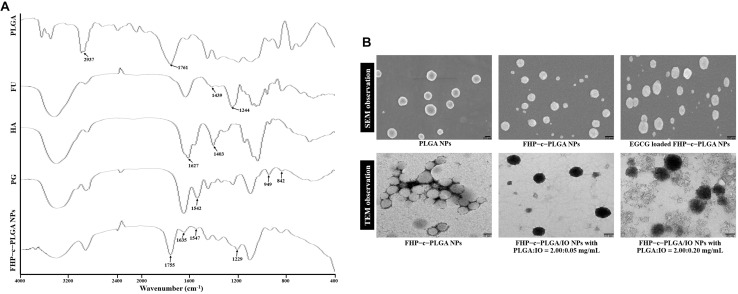
**(A)** Fourier transform infrared analyses of PLGA, FU, HA, PG, and FHP–c–PLGA NPs. **(B)** Scanning electron microscopy (SEM) or transmission electron microscopy (TEM) micrographs of the different NP preparations.

The FTIR analyses detected characteristic peaks of PLGA at 1,761 cm^–1^ and 2,937 cm^–1^ assigned to the stretching bands of C = O and C–H. The spectrum of FU showed stretching vibrations of O = S = O of sulfate esters at broad bands at approximately 1,244 cm^–1^. Scissoring vibrations of CH_2_ (galactose, xylose) or asymmetric bending vibrations of CH_3_ (fucose, O-acetyls) were detected at 1,439 cm^–1^, and HA exhibited C = O asymmetric stretching at 1,627 cm^–1^ and C–O symmetric stretching at 1,403 cm^–1^, corresponding to carboxyl groups. Symmetrical PG peaks at 949 and 842 cm^–1^ corresponded to the reflected bending of C–O and C–C stretching vibrations, respectively, of PEG, and a characteristic band at 1,542 cm^–1^ represented the bending associated –NH stretching vibration of gelatin. The spectrum of the FHP–c–PLGA complex showed characteristic bands at 1,755 cm^–1^, representing the C = O deformation band of PLGA and at 1,547 cm^–1^, representing –NH bending of PG. Moreover, the characteristic C = O stretching at 1,627 cm^–1^ of HA and S = O stretching at 1,244 cm^–1^ of FU shifted to 1,635 and 1,229 cm^–1^. These observations reflect intramolecular and intermolecular hydrogen bonding between –NH of PG to C = O of HA or PLGA (C = O⋅⋅⋅H–N) and S = O of FU (S = O⋅⋅⋅H–N; Figure 2A). Furthermore, PLGA, FU, HA, and PG complexes segregated into colloidal NPs (Figure 1).

### Characterization of EGCG-Loaded FHP–c–PLGA NPs and Drug-Release Profiles

Epigallocatechin gallate-loaded FHP–c–PLGA NPs were produced by gelation of different concentrations of EGCG solution with aqueous FHP-c-PLGA NPs solution. The EGCG loading efficiencies and loading contents were as follows: 43.98 ± 7.87% and 9.87 ± 1.69% for EGCG (0.5 mg/mL); 47.86 ± 4.57% and 19.67 ± 2.48% for EGCG (1.0 mg/mL), and 37.96 ± 6.68% and 22.17 ± 3.98% for EGCG (1.5 mg/mL). Formation with EGCG concentration 1.0 mg/mL, the sizes of EGCG-loaded NPs were 217.19 ± 11.37 nm; zeta potential values were -35.75 ± 2.39 mV, and appreciably narrower distributions (0.25 ± 0.09), which formed a spherical and uniform matrix (Table 2 and Figure 2B).

**TABLE 2:** Effect of different epigallocatechin gallate (EGCG) concentration on particle sizes, polydispersity indices, zeta potential values, and drug-loading efficiency of EGCG loaded FHP-c-PLGA NPs (*n* = 5).

EGCG (mg/mL)	Mean Particle Size (nm)	Polydispersity indices	Zeta Potential Value (mV)	Loading efficiency (%)	Loading content (%)
**FU/HA/PG concentration at 1.2:1.2:1.2 (mg/mL)**					
0.0	198.89 ± 12.56	0.22 ± 0.09	−33.75 ± 1.24	ND	ND
0.5	208.79 ± 8.78	0.24 ± 0.11	−34.89 ± 1.87	43.98 ± 7.87	9.87 ± 1.69
1.0	217.19 ± 11.37	0.25 ± 0.09	−35.75 ± 2.39	47.86 ± 4.57	19.67 ± 2.48
1.5	238.86 ± 13.27	0.29 ± 0.08	−34.97 ± 1.12	37.96 ± 6.68	22.17 ± 3.98

*NPs, nanoparticles.*

**FIGURE 3:**
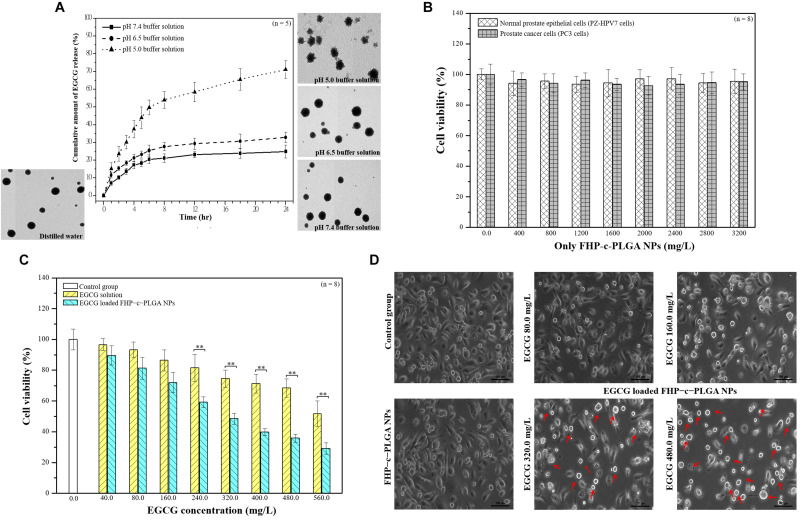
**(A)** Transmission electron microscopy (TEM) micrographs and *in vitro* profiles of the release of epigallocatechin gallate (EGCG) from EGCG-loaded FHP–c–PLGA NPs in buffers differing in pH values at 37°C. **(B)** Cytocompatibility of normal prostate epithelial cells and prostate cancer cells after treated with different concentrations of only FHP–c–PLGA NPs. **(C)** Prostate cancer cell viability was determined using 3-(4,5-dimethylthiazol-2-yl)-2,5-diphenyltetrazolium bromide (MTT) assays after cells were treated with EGCG solution or EGCG-loaded FHP–c–PLGA NPs containing different concentrations of EGCG. Asterisk ** represents statistically a significant difference of *p* value < 0.01. **(D)** Microscopic observations of cells treated with different concentrations of EGCG in EGCG-loaded FHP–c–PLGA NPs, FHP–c–PLGA NPs, or controls.

We next evaluated the pH-responsive property of EGCG-loaded NPs by using HPLC and TEM to determine drug release profiles and the morphologies of the NPs. At pH 7.4–6.5 (simulating the pH values of physiological fluids or the environment of tumor tissues), EGCG stably bound to PG to form hydrogen bonds, which generated spheres in the matrix. The percentages of EGCG released from NPs after 3 h incubation was 13.68 ± 1.73% at pH 7.4 and 18.41 ± 1.17% at pH 6.5. In contrast, at pH 5.0, –COO^–^ groups were partially protonated in HA, affecting the negative electrostatic interaction between NPs. The structures of the NPs became unstable, accompanied with gradual increases of EGCG release from 30.25 ± 3.79% at 3 h to 71.12 ± 4.96% at 24 h (Figure 3A).

### Cytotoxicities of Only FHP–c–PLGA NPs and EGCG Solution or EGCG-Loaded FHP–c–PLGA NPs

We investigated the effects of only FHP–c–PLGA NPs on the cytocompatibility of normal prostate epithelial cells (PZ-HPV7) and prostate cancer cells (PC3 cells). As shown in Figure 3B, cell viability was generally not all affected after treatment alone with FHP–c–PLGA NPs less than 3,200 mg/L. We next examined the effects of EGCG and EGCG-loaded NPs on the proliferation of prostate cancer cells. We then determined the cytotoxic effects of EGCG and EGCG-loaded FHP–c–PLGA NPs in cancer cell. Cell viability was not significantly affected by EGCG-loaded NPs and by EGCG alone at EGCG concentrations less than 40.0 mg/L and 80.0 mg/L. In contrast, both treatments significantly inhibited cell viabilities in a concentration-dependent manner. The 50% inhibitory concentration (IC50) of EGCG-loaded NPs was found to be 320.0 mg/L, in contrast to 560.0 mg/L for the EGCG solution. EGCG-loaded NPs presented better anti-cancer effects compared to EGCG alone (Figure 3C). To evaluate EGCG functions in EGCG-loaded FHP–c–PLGA NPs, the morphological changes were observed (Figure 3D). Control and FHP–c–PLGA NP-treated cells were oblong and firmly attached to the substratum in a flattened cobblestone arrangement. The cells had abundant cytoplasm and only occasional round, floating cells (dead) were observed in EGCG-loaded FHP–c–PLGA NPs treated culture. When cells were treated with EGCG-loaded NPs (EGCG 320.0 mg/mL and 480.0 mg/mL), more dramatic morphological changes were observed, and a higher percentage of cells were small. The more pronounced morphological changes (i.e., red arrows) and reduction in cell numbers were induced under these EGCG-loaded NPs treatments.

**FIGURE 4:**
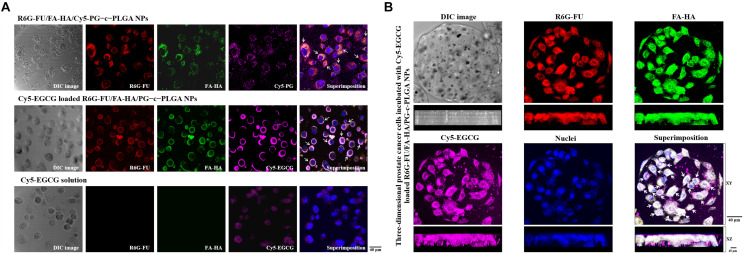
**(A)** Confocal images of prostate cancer cells showing cellular distribution of the NP preparations. **(A)** Treatment with only R6G-FU/FA-HA/Cy5-PG–c–PLGA NPs or Cy5-epigallocatechin gallate (EGCG)-loaded R6G-FU/FA-HA/PG–c–PLGA NPs vs. Cy5-EGCG. **(B)** Three-dimensional analysis of fluorescent PC3 cells adhered to Matrigel-coated microcarriers incubated with fluorescent Cy5-EGCG-loaded R6G-FU/FA-HA/PG–c–PLGA NPs.

Cellular distribution of NP complexes and evaluation of apoptosis induced by EGCG-loaded NP. The distribution of NPs in PC3 cells were detected using CLSM analysis (Figure 4A). PC3 cells were incubated with fluorescent R6G-FU/FA-HA/Cy5-PG–c–PLGA NPs (R6G-FU: red spot; FA-HA: green spot; Cy5-PG: purple spot). Fluorescence of NPs were co-localized and remained intact (as indicated by superimposed red/green/purple spots, i.e., white arrows) when internalized into the cytoplasm. Cy5–EGCG subsequent fluorescence signals emitted by Cy5-EGCG-loaded R6G-FU/FA-HA/PG–c–PLGA NPs (R6G-FU: red spot; FA-HA: green spot; Cy5-EGCG: purple spot, i.e., white arrows) were in contact with PC3 cancer cells. More EGCG was released into cytoplasm compared to EGCG solution treatment.

**FIGURE 5:**
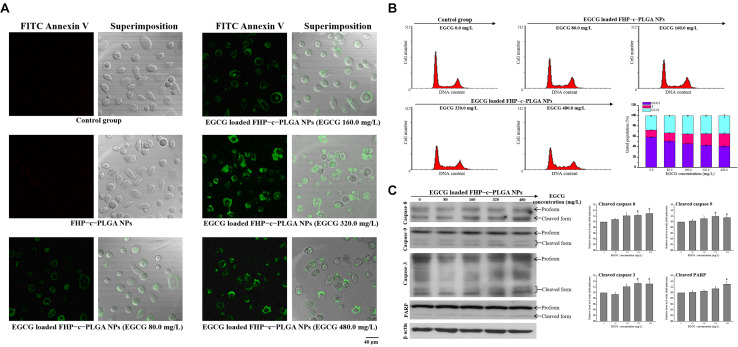
**(A)** Fluorescence detection of apoptotic prostate cancer cells (PC3) cells after treatment with FHP–c–PLGA NPs loaded with different epigallocatechin gallate (EGCG) concentrations. Controls were labeled with FITC-Annexin V and analyzed using a con-focal laser scanning microscope. **(B)** The proportions of cells in the G0/G1, S, and G2/M phases after treatment with EGCG-loaded FHP-c-PLGA NPs are indicated. The cells were stained with propidium iodide, and the cell cycle distribution was analyzed using flow cytometry. **(C)** Western blotting analysis of apoptosis-related caspase-8, -9, -3, and poly (ADP-ribose) polymerase (PARP) in PC3 cells after incubation with EGCG-loaded FHP–c–PLGA NPs, and β-actin was used as an internal control. Asterisk * represents statistically a significant difference of *p* value < 0.05, as compared with the without sample treatment group.

The three-dimensional PC3 cells were incubated with fluorescent EGCG-loaded NPs. CLSM analyses showed the superimposition of the images produced by Cy5-EGCG-loaded R6G-FU/FA-HA/PG-c-PLGA NPs (as indicated by superimposed green/red/purple spots, i.e., white arrows), which co-localized and interacted locally along with the *XY* plane and appeared deep in the *XZ* plane (Figure 4B). Higher EGCG concentration of EGCG-loaded NPs treatment, significantly reduced cell viability and induced morphological changes from oblong to coccoid forms after 24 h, which correlated with FITC-Annexin V expression (i.e., green spots; Figure 5A). The binding of Annexin V is regarded as an apoptotic indicator according to the interaction of Annexin V to the inner cell membrane component phosphatidylserine. The fluorescence of FITC-Annexin V conjugate was undetectable in controls or FHP–c–PLGA NP-treated cells. In contrast, incubation of EGCG-loaded NPs led to induce the expression of green fluorescence at cell surfaces in a concentration-dependent manner.

### Effects of EGCG-Loaded NPs on Cell Cycle Arrest and Apoptosis

To determine whether EGCG-loaded FHP-c-PLGA NPs arrest the cell cycle progression, we treated PC3 cells with propidium iodide following NP treatments with different concentrations of EGCG. EGCG induced a significant decrease of G0/G1 populations (decreased from 59.9 ± 1.34 to 41.13 ± 1.96%), an increase in cells undergoing S phase (increased from 13.04 ± 0.98 to 24.38 ± 2.31%), and the accumulation of cells in G2/M (increased from 27.87 ± 0.91 to 34.49 ± 2.87%; Figure 5B). To determine whether EGCG-loaded NPs induce apoptosis, we performed western blotting to determine the ratios of cleave-caspase-8, caspase-9, caspase-3, and PARP to their total (cleaved form plused proform) protein expressions. Twenty-four hours after EGCG-loaded NPs treatment, EGCG (480 mg/mL) increased the ratios of cleaved to total expressions of caspase-8 and -9 about 29% (caspase-8) and 15% (caspase-9) compared to the controls. The levels of cleaved caspase-3 and PARP in PC3 cells significantly increased in the presence of EGCG-loaded NPs compared with the controls (*p* < 0.05; Figure 5C).

### Molecular Imaging of NPs in Tumor Xenografts

The effects of FHP–c–PLGA/IO NPs were visualized using MRI. FHP–c–PLGA/IO NPs were prepared with different concentrations of IO. The mean particle size, homogeneity/polydispersity indices were varied with IO concentrations (Table 3). The mean sizes of these NPs ranged from 230 to 600 nm, with homogeneity/polydispersity indices that varied relative to the concentrations of IO (Table 3). Furthermore, TEM observations revealed that single FHP–c–PLGA NPs were smooth and round. As expected, the FHP–c–PLGA/IO NPs obtained using PLGA:IO at a 2.00:0.20 mg/mL were unevenly disbursed and had a larger average diameter (598.90 ± 49.45 nm) compared to NPs obtained using PLGA:IO at 2.00:0.05 mg/mL, which exhibited an internal structure consisting with black granular IOs dispersed among gray NPs (Figure 2B).

**TABLE 3:** Effect of different PLGA/IO concentrations on particle sizes, polydispersity indices, and zeta potential values of the FHP-c-PLGA/PLGA/IO NPs (*n* = 5).

PLGA:IO (mg/mL)	Mean Particle Size (nm)	Polydispersity indices	Zeta Potential Value (mV)
**FU/HA/PG concentration at 1.2:1.2:1.2 (mg/mL)**			
2.00:0.05	232.64 ± 13.87	0.28 ± 0.04	−36.76 ± 2.47
2.00:0.10	319.57 ± 19.68	0.39 ± 0.12	−38.52 ± 5.91
2.00:0.20	598.90 ± 49.45	0.72 ± 0.27	−37.52 ± 2.35

*PLGA, poly (D,L-lactide-co-glycolide); NPs, nanoparticles.*

**FIGURE 6:**
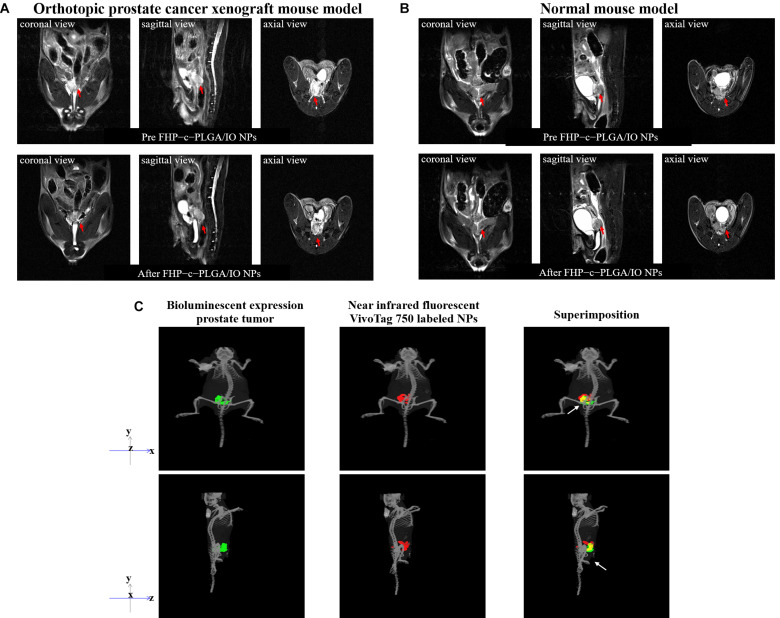
*In vivo* T2-weighted axial MR images were acquired before and after injection of FHP–c–PLGA/IO NPs from untreated and xenografted mice. A decrease in signal intensity caused by FHP–c–PLGA/IO NPs was observed in prostate tumor tissue (red arrows) but not in normal prostate tissue. **(A)** Orthotopic prostate cancer xenograft mouse model. **(B)** Normal mouse model. **(C)** Three-dimensional bioluminescence or fluorescence optical tomography using *in vivo* imaging system (IVIS) Spectrum CT. The co-localization (yellow) of bio-luminescent prostate tumors (green) with VivoTag 750-labeled NPs (red) in the prostate tissue (white arrows).

The proportion of PLGA/IO at 2.00:0.05 produced the NPs with minimum mean particle size (232.64 ± 13.87 nm) and uniform polydispersity index (0.28 ± 0.04) and were therefore used for MRI analyses. FHP–c–PLGA/IO NPs achieved significant, concentration-dependent MRI contrast-enhancement (Figure 1). The MR imaging appeared hypointense as the IO concentration increased, i.e., the shorter T2-relaxation time. The MR images obtained using the T2W sequence before and after injection of mice with FHP–c–PLGA/IO-labeled NPs were shown in Figures 6A,B. The hypointensity of prostate tumor was clearly visualized in the T2W MR image because of the T2-shortening effect caused by the IOs (i.e., red arrows; Figure 6A). In contrast, in the control group, the MRI signals detected at the prostate were not significantly different before and after injection of sample (Figure 6B). Moreover, target-specific near infrared fluorescent VivoTag 750-labeled NPs were injected into the tail veins of Luc-PC3 tumor-bearing mice. The IVIS Spectrum CT images of Luc PC3 tumor-bearing mice were acquired after injection of VivoTag 750-labeled NPs. The axial image clearly showed the dominant accumulation of fluorescent NPs signals in the region of the prostate tumor (as indicated by superimposed green/red spots; i.e., yellow spots, white arrows; Figure 6C).

### Assessment of Antitumor Activity and Histological Analysis

**FIGURE 7:**
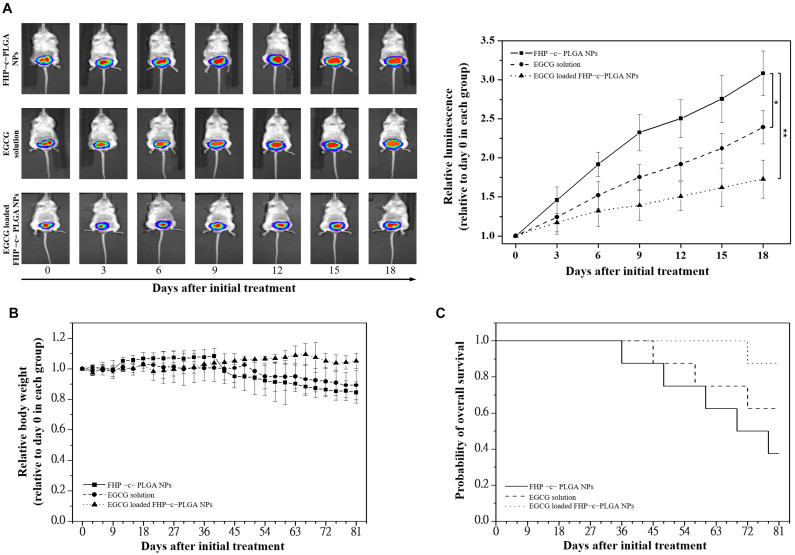
Antitumor effects of epigallocatechin gallate (EGCG)-loaded FHP–c–PLGA NPs in orthotopic Luc prostate cancer cells (PC3) xenograft mice models. Mice were divided into three groups of six mice and were treated with only FHP–c–PLGA NPs (■), EGCG solution (●), or EGCG-loaded FHP–c–PLGA NPs (▲) every 3 days for 21 days. **(A)** Antitumor activities using a noninvasive *in vivo* imaging system. Asterisk *, ** represents statistically significant difference of *p*-value < 0.05, and *p*-value < 0.01, respectively. **(B)** Changes in relative body weight. **(C)** Kaplan–Meier survival curves of mice bearing orthotopic Luc-PC3 xenografts.

We next investigated the tumor-specific effects of EGCG-loaded NPs in the orthotopic mouse model of prostate cancer. The prostate tumor bioluminescence signals significantly increased with time by 3.08 ± 0.29- and 2.39 ± 0.21-fold at day 18 in mice treated with FHP–c–PLGA NPs and EGCG, respectively (Figure 7A). In contrast, the growth of prostate tumors was significantly inhibited in mice treated with EGCG-loaded FHP–c–PLGA NPs, with a lower relative photon flux compared with the other groups of treated mice. There was no significant difference in the loss of body weight between groups (Figure 7B). The survival rates of mice treated with EGCG-loaded NPs were significantly longer compared with controls, indicating significant antitumor activity (Figure 7C).

**FIGURE 8:**
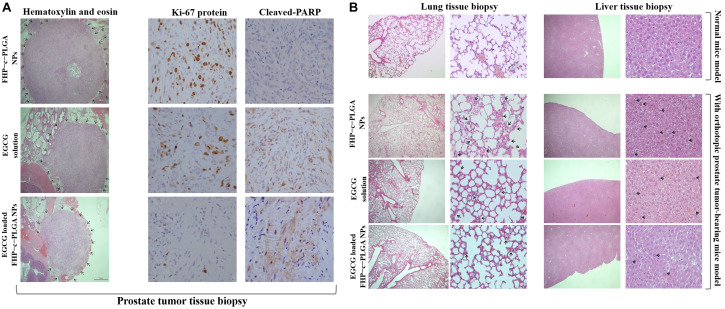
Histological and immunohistochemical analyses of orthotopic prostate tumors treated with FHP c PLGA NPs, EGCG solution, or EGCG-loaded FHP c PLGA NPs. **(A)** Prostate biopsy. The black arrows indicate the tumor range. **(B)** Lung and liver biopsies. The black arrows indicate the inflammation.

In the control group (treated with FHP–c–PLGA NPs), tumor-bearing prostate gland tissue had well-defined and unencapsulated margins, with the largest tumor range and foci of tumor cells in the surrounding stroma (i.e., black arrows, Figure 8A). Immunohistochemical examination revealed that the expression of cell proliferation marker Ki-67 was reduced and the expression of apoptotic marker (cleaved PARP) was increased in tumors treated with EGCG-loaded FHP–c–PLGA NPs (i.e., coffee dots, Figure 8A). Prostate cancer may induce in systemic inflammation *in vivo*. In the present study, the lung tissues of the normal mice were clear and intact, with no infiltration of inflammatory cells, bleeding, edema, or thickening of the alveolar wall. The mice with prostate tumors in control group had disorganized alveolar structures, significant exudation of inflammatory cells, red blood cells dispersed in multiple alveolar cavities, and interstitial pulmonary edema and thickening (i.e., black arrows, Figure 8B). The pathological damage was significantly milder in the EGCG-loaded NPs group that exhibited clear alveolar structures, insignificant interstitial pulmonary edema and thickening, and reduced inflammation (i.e., black arrows, Figure 8B). Hepatocyte swelling and neutrophil infiltration were decreased in liver tissues of mice treated with EGCG-loaded NPs compared to control mice. It is suggested that targeting NPs could not only significantly increase EGCG’s antitumor activity against prostate tumors but also reduce the inflammatory reaction in the body.

## Discussion

Targeted therapy that delivers drugs to cancer cells is important for improving treatment efficacy and avoiding systemic toxicity (Li et al., 2020; Shen et al., 2020). In recent years, the focus on developing targeted therapy with NP delivery systems has increased (Swain et al., 2016; Genchi et al., 2017; Adekiya et al., 2020). Molecular targeting relying on binding between a targeting ligand and a cancer-specific receptor has been used extensively in nanomaterials techniques (Yao et al., 2016). In the present study, using a targeted NPs system, we detected significant increases in the fluorescence intensities of Rh6G-FU/FA-HA-complexed NPs, which emitted detectable fluorescence and did not interfere with each other when taken up by prostate cancer cells (Figure 4). FU represents a class of sulfated fucose-rich polysaccharides synthesized from brown algae. Prostate-specific membrane antigen, which contains fucosylated oligosaccharides, is an important marker of prostate cancer, and its level is increased many folds in prostate cancer and in the neovasculature of other tumors (Fitton et al., 2003; Ghosh and Heston, 2003; Synytsya et al., 2010). HA, which is a major component of the vertebrate extracellular matrix, efficiently targets CD44 through hydrogen bonds and van der Waals interactions (Banerji et al., 2007). We found that the EGCG-loaded NPs attached to PC3 cells. Furthermore, the carrier EGCG induced S phase or G2/M cell-cycle arrest, which is involved in inhibition of PC3 cell proliferation, and enhanced the expression of apoptosis-related proteins (Figure 5).

Integrated diagnosis and therapy systems that can offer effective cancer therapy are in high demand for personalized medicine. Molecular imaging techniques such MRI, CT, ultrasound, and fluorescence microscopy play important roles in medicine and biomedical research (Kherlopian et al., 2008; Thorat et al., 2019). Here, NPs containing IOs were intravenously injected into the tail veins of mice. MRI revealed that these NPs were retained at the site of prostate tumors compared with the control group, and there were no significant differences in T2W intensities between test and control mice (Figures 6A,B). In this study, we demonstrated that NPs containing IOs can be targeted to the prostate tumor instead of the normal prostate tissue. Every imaging technique has its own limitations, such as insufficient sensitivity, spatial resolution, data acquisition time, and complexity, which hinder the acquisition of accurate information. Multi-imaging modalities overcome these shortcomings (Zhao et al., 2018). For example, three-dimensional bioluminescence or fluorescence optical tomography using IVIS Spectrum CT clearly showed that VivoTag 750-labeled NPs mainly accumulated in the region of the prostate tumor and that NPs targeted the tumor with accurate detection (i.e., yellow spots, white arrows, Figure 6C).

Epigallocatechin gallate-loaded NPs with optimal pH responsiveness can improve the delivery and control the drug locally targeting to the tumor site. This type of NP delivery system is under intensive study. The pH value of tumor tissues (pH 6.5) differs from that of healthy tissues (pH 7.4). Furthermore, the pH value of intracellular endosomes and lysosomes ranges from pH 5.0 to 6.0. Endosomes and lysosomes trigger the drug’s release from designed NPs into cancer cells. The EGCG-loaded NPs studied here were designed with a matrix structure for minimizing drug release into normal physiological environments such as blood and non-target tissues (pH 6.5–7.4). Such NPs facilitated the release of EGCG from late endosomes and lysosomes of tumor cells (cumulative release, 71.12 ± 4.96% at pH 5.0; Figure 3A). Specifically, *in vivo* experiments confirmed that significant enhancement of the inhibition of tumor growth was demonstrated by the lower bioluminescent intensity after intravenous EGCG-loaded NPs (15.0 mg/kg EGCG; Figure 7). Moreover, histological analyses demonstrated that the delivery of EGCG to tumors by NPs induce apoptosis in PC3 cells as indicated by increased levels of cleaved PARP accompanied by a reduction in inflammatory pulmonary lesions (Figure 8). Further preclinical and clinical studies will be of paramount importance to further enhance our understanding of the potential of such NPs as alternative anticancer treatments.

## Conclusion

The present study indicated that NPs with combined therapeutic and molecular imaging attributes can effectively target prostate cancers, leading to significant enhancement of anti-prostate tumor activity, as shown by the localization of tumors in an orthotopic mouse model of prostate cancer. These theranostic NPs have the potential to provide a delivery system for identifying alternative anticancer treatments and new diagnostic techniques that will facilitate clinical trials.

## Data Availability Statement

The original contributions presented in the study are included in the article/supplementary material, further inquiries can be directed to the corresponding author.

## Ethics Statement

The animal study was reviewed and approved by Institutional Animal Care Use Committee (Approval No. 1061213), National Yang-Ming University, Taiwan.

## Author Contributions

S-LP, C-HL, and P-YC: conception or design of this work. S-CC: experimental study. J-TH and Y-CT: data analysis and interpretation. Y-HL: wrote the manuscript and obtained funding. All authors read and approved the manuscript.

## Conflict of Interest

The authors declare that the research was conducted in the absence of any commercial or financial relationships that could be construed as a potential conflict of interest.
